# Effect of Glass Fiber-Reinforced Plastic Waste on the Mechanical Properties of Concrete and Evaluation of Its Feasibility for Reuse in Concrete Applications

**DOI:** 10.3390/ma16206772

**Published:** 2023-10-19

**Authors:** Tianhao Zhao, Yong Lv, Jianzhong Chen, Pengfei Song, Mingqing Sun, Xiaoyu Zhang, Li Huang

**Affiliations:** 1 Hubei Key Laboratory of Theory and Application of Advanced Material Mechanics, School of Science, Wuhan University of Technology, Wuhan 430070, China; thob06@163.com (T.Z.);; 2 Hengrun Group New Materials Co., Ltd., Hengshui 053100, China

**Keywords:** glass fiber-reinforced plastic (GFRP), concrete, mechanical properties, mechanical recycling, optimized reuse process

## Abstract

The disposal of glass fiber-reinforced plastic (GFRP) waste has become an urgent issue in both the engineering and environmental fields. In this study, the feasibility of reusing mechanically recycled GFRP in concrete was evaluated. Secondary screening of the recycled material was conducted to obtain different types of products, and the recycled GFRP (rGFRP) was characterized. Subsequently, the effect of rGFRP on concrete performance was evaluated using different dosages (0%, 1%, 3%, 5%) of rGFRP powder and rGFRP cluster (with different sizes and fiber contents) to replace fine aggregate in concrete preparation. The experimental results indicated that the addition of rGFRP powder has no significant impact on the mechanical properties of concrete, while the addition of a small amount of rGFRP cluster slightly improves the compressive strength and splitting tensile strength of concrete. Additionally, the short fibers in rGFRP improve the failure mode of concrete, and increased fiber content and longer fiber length demonstrate a more pronounced reinforcing effect. The challenges and potential directions for future research in the realm of reusing rGFRP in concrete are discussed at the end. A systematic process for reusing GFRP waste in concrete is proposed to address the primary challenges and provide guidance for its practical engineering application.

## 1. Introduction

In recent years, the use of glass fiber-reinforced plastic (GFRP) has been widely adopted in the aerospace, construction, and automotive industries [1,2,3,4,5,6]. However, as the production and usage of GFRP products continue to rise, a significant amount of GFRP waste is expected to reach the end of its service life in the future [7,8]. Additionally, a considerable amount of GFRP waste is generated during the production and processing of GFRP products, posing a significant waste challenge [9]. Therefore, the efficient and cost-effective management of GFRP waste has gained increasing attention.

Many countries have imposed restrictions and prohibitions on traditional landfilling and incineration methods [10]. Therefore, it is crucial to develop innovative GFRP waste management approaches. Previous studies have explored the chemical and thermal recycling of GFRP. However, due to the high recycling costs and the complexity of recycling processes, thermal and chemical recycling may not be the most suitable options for GFRP [11,12,13,14,15,16].

At present, mechanical recycling has emerged as the most practical and feasible method for managing GFRP waste due to its low cost, minimal environmental impact, and capability to efficiently process large quantities of GFRP waste [17]. The mechanical recycling process involves a series of steps, including sorting, cutting, grinding, and screening, aimed at reducing the size of GFRP waste [18,19].

The construction industry has made significant efforts to reduce waste and promote recycling to mitigate adverse environmental impacts [20,21,22,23]. Using recycled GFRP (rGFRP) obtained through mechanical recycling to produce concrete seems to be a promising solution. This approach not only reduces the necessity for landfilling such materials, but also preserves acceptable concrete quality, in some cases even higher [24,25,26,27].

Asokan et al. [28] found that the addition of GFRP waste powder improved the mechanical properties of concrete, despite a slightly higher w/c ratio. Correia et al. [29] used GFRP waste particles to partially replace the fine aggregate in concrete. They found that when the recycled GFRP content was high, there was a significant decrease in all mechanical properties. García et al. [30] optimized the recycling process and used the obtained rGFRP material to prepare microconcrete specimens. They found that adding 1% rGFRP improved the compressive strength of the microconcrete specimens. Tittarelli et al. [31] used GFRP powder from a shipyard as an industrial by-product and replaced 5% and 10% of the natural sand. They found that the compressive strength of the concrete significantly decreased by about 40% and 50%, respectively. Dehghan et al. [32] investigated the effect of rGFRP on the mechanical properties of accelerated mortar and found that there was no significant improvement in compressive strength, but an increase in splitting tensile strength.

The results from the previously mentioned studies reveal significant differences. Even when the amount of GFRP waste added is similar, the impact on concrete’s mechanical properties varies distinctly. This variation can be attributed to (1) differences in GFRP waste size, including particle size and fiber length, resulting from various recycling processes, and (2) differences in the composition of GFRP waste from different sources, particularly in terms of fiber content. Therefore, it is necessary to systematically evaluate the effect of these factors on concrete performance.

In this study, an experimental study was conducted to investigate the effects of mechanically recycled GFRP with different sizes, compositions, and contents on the performance of concrete, and to evaluate its feasibility of reuse in concrete. Subsequently, the challenges faced by reusing GFRP waste in concrete at the current stage were analyzed, and existing reuse methods were summarized and optimized, enabling us to propose a systematic reuse program.

## 2. Materials and Methods

### 2.1. Material Origin and Components

**Figure 1:**
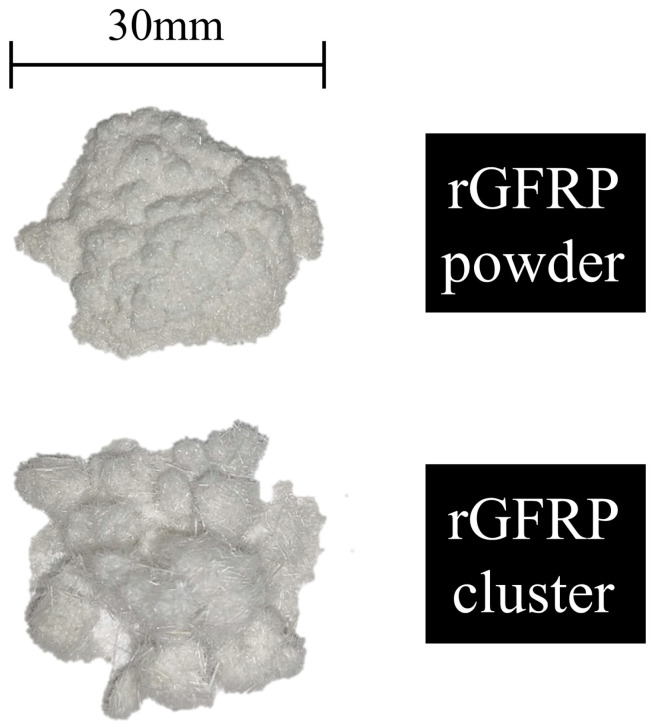
rGFRP powder and rGFRP cluster.

**Figure 2:**
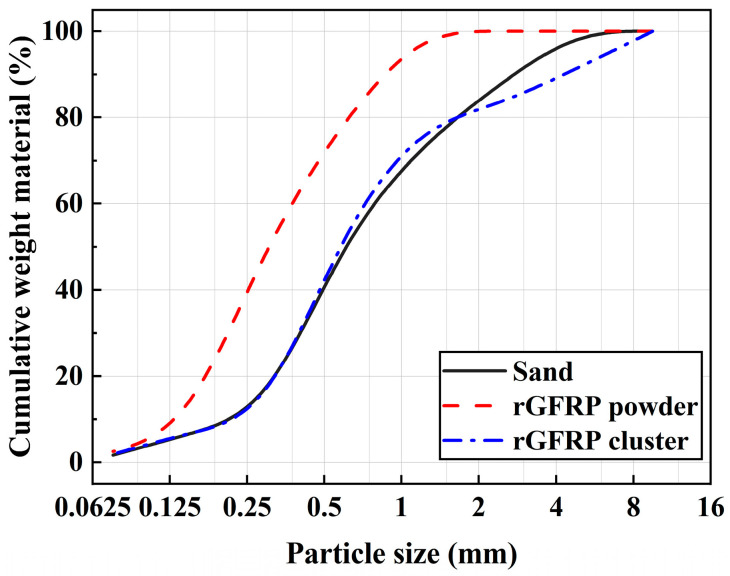
Particle size distribution of rGFRP and sand.

The rGFRP used in this study was mechanically recycled from decommissioned wind turbine blades. GFRP material is composed of E-glass fiber and unsaturated polyester resin. After the GFRP waste was crushed and sieved, secondary screening using a 1.18 mm sieve was conducted to obtain two types of recycled materials, namely, rGFRP powder and rGFRP cluster. As shown in Figure 1, the rGFRP powder had a granular form with a considerable amount of resin dust, while the rGFRP cluster took on a fluffy cluster form. Concrete specimens were prepared using P.O. 42.5 cement. Crushed limestone with a maximum particle size of 20 mm was used as a coarse aggregate. The crushing index of gravel is 16%, meeting the requirements of GB/T 14685-2022 [33]. Natural river sand with a fineness modulus of 2.68 was used as the fine aggregate. Figure 2 shows the grading curves of the rGFRP and sand. To improve the concrete properties, polycarboxylate superplasticizer (1.5% of cementitious materials content), fly ash, and mineral powder were added to all concrete. The polycarboxylate superplasticizer has a water-reducing rate of 25% and a solid content of 19%. The chemical compositions of cement, fly ash, mineral powder, and rGFRP were determined using X-ray fluorescence spectroscopy (Malvern Panalytical Zetium, Almoro, The Netherlands) via the pellet method and are listed in Table 1. All the raw materials used complied with GB 50164 [34].

### 2.2. Concrete Mix Proportions

Seven different concrete mixes were used in this study, as outlined in Table 2. The first mix, labeled as CC, was a control mix containing no rGFRP. The remaining six mixes, labeled as P01, P03, P05, C01, C03, and C05, contained varying amounts of rGFRP powder or cluster, replacing 0%, 1%, 3%, and 5% of the fine aggregate. The only variable across all the mixes was the substitution rate of rGFRP to investigate the effect of rGFRP on concrete properties.

### 2.3. Specimen Preparation and Testing Procedures

**Figure 3:**
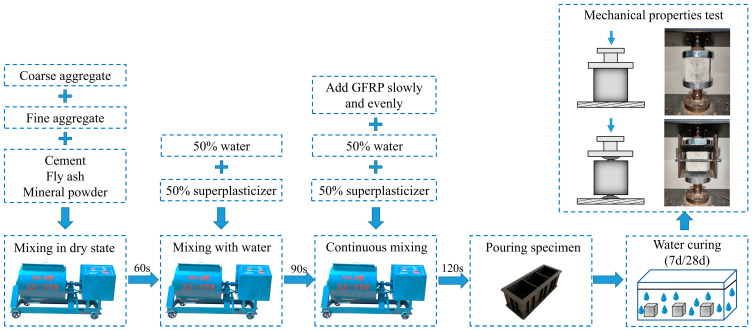
Preparation and testing process of rGFRP concrete specimen.

The process of specimen preparation and testing is outlined in Figure 3. To prepare the concrete specimens, a single-shaft horizontal mixer was used. The first step involved the sequential addition of the coarse aggregate, fine aggregate, and cementitious material into the mixer, followed by dry mixing for one minute. Subsequently, 50% of the required water and superplasticizer were added, followed by mixing for 1.5 min. Finally, the remaining water and superplasticizer were added, and mixing continued for an additional 2 min. During this mixing process, the rGFRP was gradually added to ensure its thorough dispersion throughout the concrete.

Following the mixing process, the fresh concrete was tested for slump according to the standards outlined in GB/T 50080 [35]. The concrete was then poured into molds, and all specimens were demolded after 24 h of casting. To ensure consistency, all specimens were cast from the same concrete batch. They were then cured by being immersed in a saturated Ca(OH)_2_ solution at room temperature until the testing day. For each type of concrete, a total of twelve cubes, measuring 100 mm × 100 mm× 100 mm, were prepared.

After the concrete attained the appropriate curing age, a series of performance tests were conducted, including mechanical property and density tests, on the hardened concrete. A summary of the test specifications and specimen sizes can be found in Table 3.

The composition of rGFRP was analyzed using a simultaneous thermal analyzer (NETZSCH STA449F3 Jupiter, Selb, Germany). Additionally, the microscopic morphology of rGFRP was characterized using scanning electron microscopy (JEOL JSM-IT300, Tokyo, Japan).

## 3. Results

### 3.1. Test on the rGFRP

**Figure 4:**
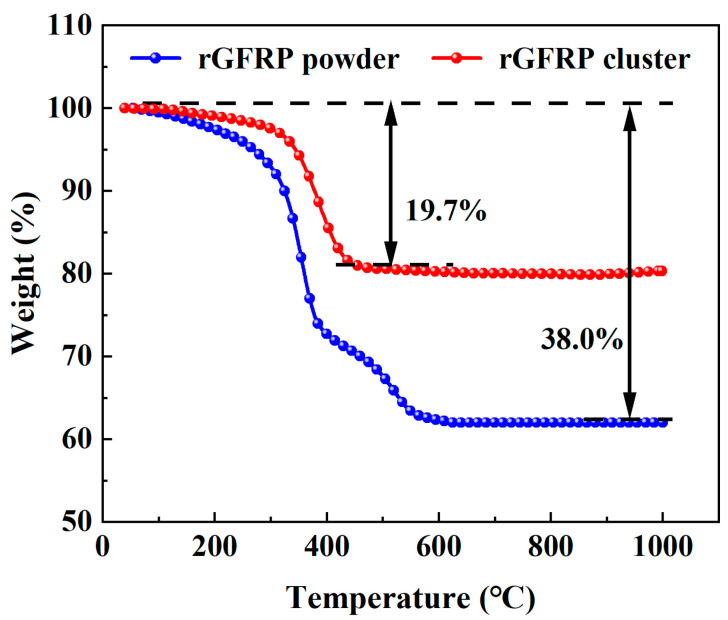
Comparison of mass loss of two types of rGFRP.

The mass loss curves for the rGFRP are depicted in Figure 4. The trend of mass change with temperature was similar for both recycled materials until the temperature reached 400 °C. The initial mass loss from room temperature to 260 °C was primarily attributed to water evaporation. From 260 °C to 400 °C, rapid mass loss occurred due to the decomposition and combustion of the resin. However, a notable difference between the two materials was observed: the rGFRP cluster stabilized in mass after 400 °C, whereas the mass loss rate of the rGFRP powder significantly decreased from 400 °C to 600 °C, attributed to the burning of residual carbon. Beyond 600 °C, the mass loss rate decelerated, and the curve plateaued. The rGFRP powder experienced a mass loss of approximately 38%, while the rGFRP cluster experienced a mass loss of around 20%. The thermogravimetric analysis revealed that the rGFRP powder consisted of approximately 72% glass fiber and 38% organic materials, while the rGFRP cluster contained roughly 80% glass fiber and 20% organic materials.

**Figure 5:**
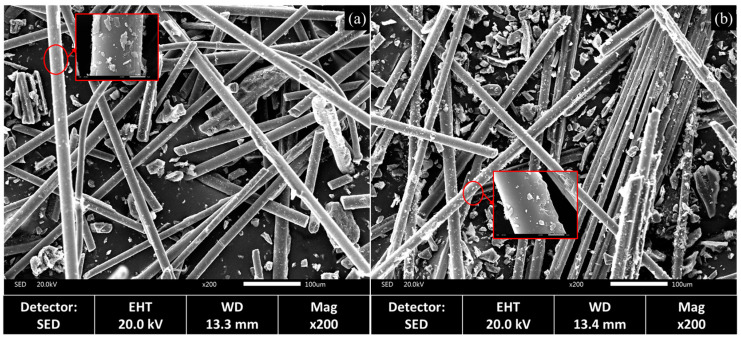
Microstructure comparison of different types of rGFRP: (**a**) rGFRP powder, (**b**) rGFRP cluster.

The microscopic morphology of rGFRP is shown in Figure 5. The analysis indicated the presence of a significant number of short fibers in both recycled materials, primarily in the monofilament form, with lengths ranging from 50 μm to 1000 μm. A few fibers appeared in bundles consisting of several fiber monofilaments. Some fibers had damaged resin layers, and resin shedding was evident, leading to the observation of a substantial amount of flaky and granular resin. Notably, the average fiber length in the rGFRP cluster was longer than that in the rGFRP powder, owing to the disparity between the two recycled materials after sieving. Additionally, more fibers were detected in the SEM images of the rGFRP cluster, which is consistent with the findings from the thermogravimetric analysis.

Based on the characterization, it is evident that the two recycled materials exhibit varying proportions of glass fibers and organic resins. Additionally, distinctions are observed in particle size (powdered component) and fiber length (fibrous component).

### 3.2. Test on the Concrete

#### 3.2.1. Effect of rGFRP on the Workability of Fresh Concrete

The workability of concrete is a crucial performance indicator that depends on the amount of water added during concrete mixing. This study compared the slump of different test groups to examine the influence of rGFRP type and content on concrete workability. All test groups had the same w/c ratio, ensuring uniform water content during concrete mixing.

**Figure 6:**
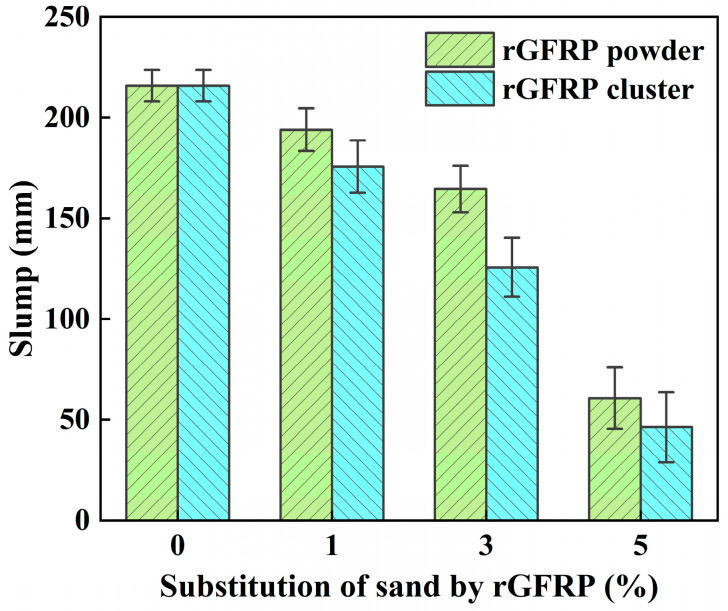
Effect of rGFRP on the slump of fresh concrete.

Before casting the test specimens, slump tests were conducted on each mixture. The slump was measured for six different batches of concrete for each mixture to reduce errors caused by manipulation or other factors. The test results are shown in Table 4 and Figure 6.

The slump of the concrete decreased linearly with an increase in rGFRP content. This outcome aligns with findings by other researchers, who have found that the high specific surface area of rGFRP, compared to natural sand, necessitates a larger surface to be covered by a water film during mixing. This leads to a decrease in free water in fresh concrete and an increase in the water demand for concrete [38]. From a rheological perspective, the presence of short fibers in rGFRP elevates the yield stress and plastic viscosity of fresh concrete [39], contributing to a reduction in slump. Furthermore, the SEM images of rGFRP also unveiled the presence of numerous irregularly shaped and sized resin particles, which could have an adverse impact on workability.

Additionally, the negative effect of rGFRP clusters on the concrete slump was more pronounced due to their longer fiber length and higher fiber content, leading to higher yield stress and plastic viscosity of fresh concrete.

#### 3.2.2. Effect of rGFRP on the Compressive Strength of Concrete

The compressive strength of CC and rGFRP concrete cube specimens was tested after 7 and 28 days of curing using a universal testing machine (MTS CMT5106, Shanghai, China), following the procedures specified in GB/T 50081 [36]. The specimens were subjected to a displacement loading rate of 0.5 mm/min and loaded until the concrete failed. Each set of experiments included three concrete samples to ensure the reproducibility of the results.

**Figure 7:**
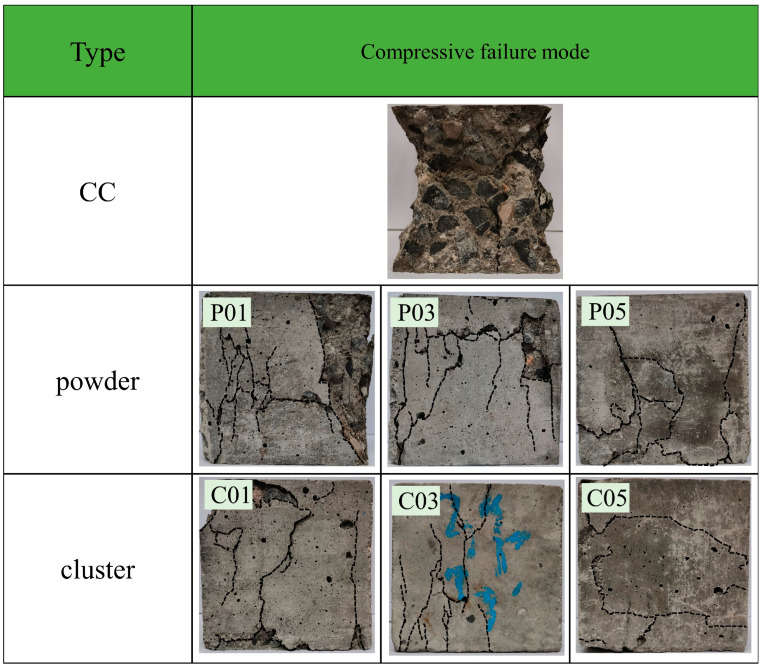
Compressive failure modes of rGFRP concrete specimens.

The compressive failure mode of the concrete is shown in Figure 7. The control concrete (CC) failure mode is characterized by large pieces of concrete peeling off and wide cracks penetrating the entire cross-section of the concrete, resulting in a conical failure pattern. In contrast, the failure mode of rGFRP concrete is characterized by multiple cracks, fewer large pieces of concrete peeling off, and a complete failure pattern. The short fibers in rGFRP restricted the initiation and propagation of some cracks, constrained the relative slip on the crack surface, and made the failure process smoother. This positive effect was more noticeable with an increase in the content of rGFRP. Due to its higher fiber content and longer fiber length, the rGFRP cluster exhibited stronger crack resistance compared to rGFRP powder.

**Figure 8:**
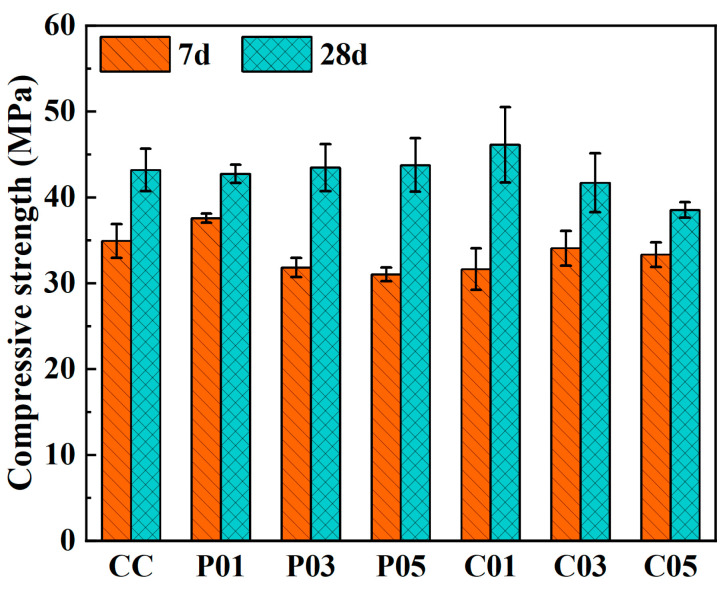
Effect of rGFRP on the compressive strength of concrete.

The results of the compressive strength tests are shown in Table 5 and Figure 8. The 7-day compressive strength of rGFRP powder concrete initially increased, and then, decreased with increasing rGFRP content. Among all the groups, the 7-day compressive strength of P01 was the highest, measuring 37.6 MPa, a 7.7% increase compared to the control group (34.9 MPa). On the other hand, the 7-day compressive strengths of the C01, C03, and C05 groups were slightly lower than that of the control group, with C01 having the lowest value of 31.6 MPa, representing a decrease of 9.5%.

The 28-day compressive strength values of concrete containing rGFRP powder were similar to those of control concrete without rGFRP (43.2 MPa), indicating that rGFRP powder had no significant effect on the 28-day compressive strength of concrete. Conversely, the influence of the rGFRP cluster on the 28-day compressive strength of concrete was more noticeable. With its content of 1%, the 28-day compressive strength of concrete was 46.1 MPa, representing a 6.8% increase. However, when the content was further increased, the compressive strength decreased. Specifically, the compressive strength decreased from 41.7 MPa to 38.5 MPa as the content was increased from 3% to 5%.

These phenomena can be attributed to several factors: (1) The presence of CaO, Al_2_O_3_, SiO_2_, and other polymers in rGFRP can strengthen the compressive strength of concrete, and the short fibers in rGFRP can inhibit the development of concrete cracks [40,41]. (2) The irregular size and shape of rGFRP may affect its bonding with other concrete components. (3) During the crushing of GFRP, some of the organic resin attached to the fibers is stripped off, as evident in SEM images. These resin particles have a lower specific modulus and transmit and resist significantly lower stresses in the concrete compared to other components [42], leading to more severe stress concentrations. When the first effect predominates, the compressive strength increases; otherwise, it decreases significantly.

#### 3.2.3. Effect of rGFRP on the Splitting Tensile Strength of Concrete

**Figure 9:**
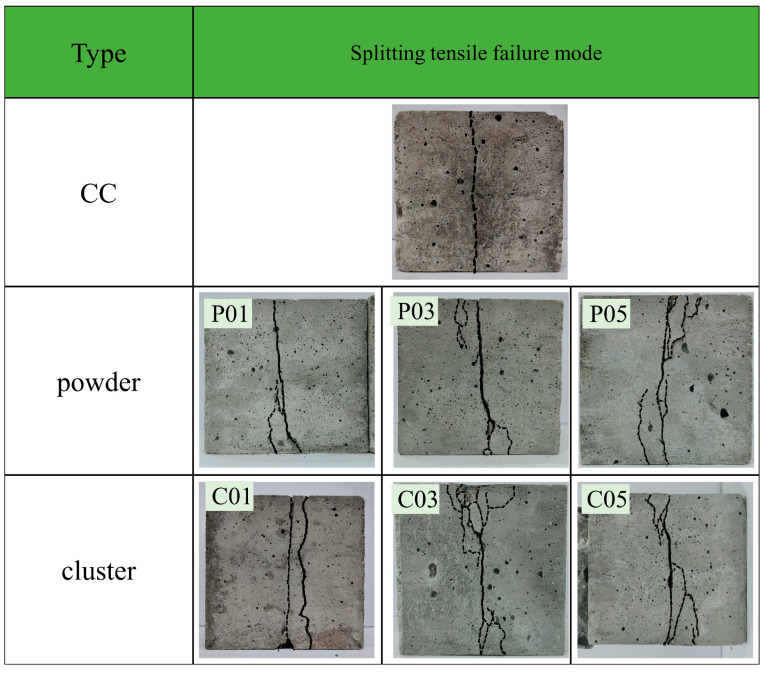
Splitting tensile failure modes of rGFRP concrete specimens.

Splitting tensile tests were conducted on concrete cube specimens according to GB/T 50081 [36]. A strip was positioned between the upper and lower circular spacers and the specimen to evenly distribute the applied load. Uniformly distributed pressure was applied to the central plane of the specimen to generate approximately uniform tensile stress in the vertical plane of the external force. The equivalent splitting tensile strength $f_{ts}$ was calculated using the following equation:

(1) $$f_{ts}=\frac{2F}{\pi A}=0.637\frac{F}{A}$$

where *A* is the area of the splitting surface of the specimen, and *F* is the maximum load recorded at the time of concrete failure. The concrete splitting tensile failure mode is shown in Figure 9.

The control group specimen exhibited single crack initiation under tensile load. As the load increased, the crack initially initiated at the center of the cross-section of the specimen, and then, propagated towards the edge strip. However, concrete specimens containing rGFRP powder and rGFRP cluster exhibited multi-crack initiation with similar failure modes. In addition to the main through-crack located at the center of the section, numerous branch cracks also existed. These branch cracks were shorter in length and did not traverse the entire surface of the concrete. The addition of more rGFRP resulted in a more pronounced multi-crack morphology. This indicated that the anisotropic microstructure of material properties, caused by the short fibers in rGFRP, changed the stress distribution and crack expansion direction. This dispersed crack expansion plays a restraining role in the main crack expansion.

**Figure 10:**
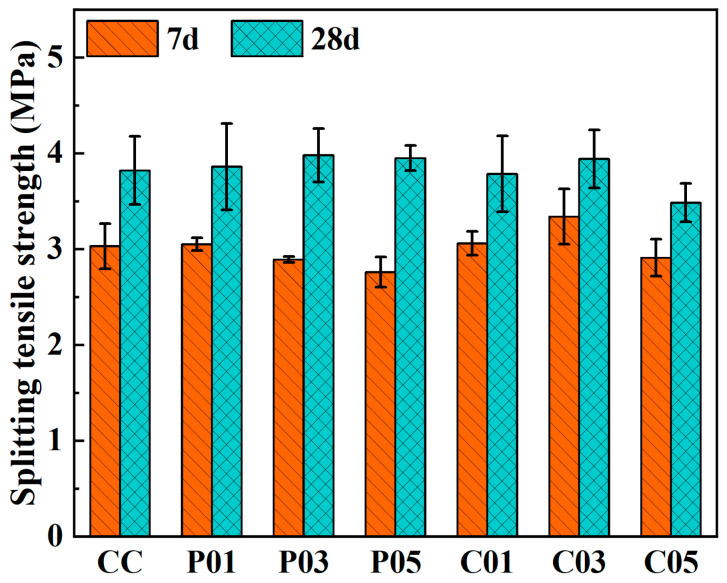
Effect of rGFRP on the splitting tensile strength of concrete.

As shown in Table 6 and Figure 10, the incorporation of rGFRP did not lead to a significant improvement in the splitting tensile strength of the concrete, contrary to expectations. Even when the concrete was incorporated with the rGFRP cluster containing more and longer fibers, the 7d and 28d splitting tensile strength of the concrete did not show a significant increase. The 28d splitting tensile strength of concrete reached its maximum value when the content of both rGFRPs was 3%, which was 4.0% and 3.1% higher than that of the control group (3.8 MPa), respectively. As the rGFRP content increased, the splitting tensile strength of rGFRP concrete tended to increase first, and then, decrease.

**Figure 11:**
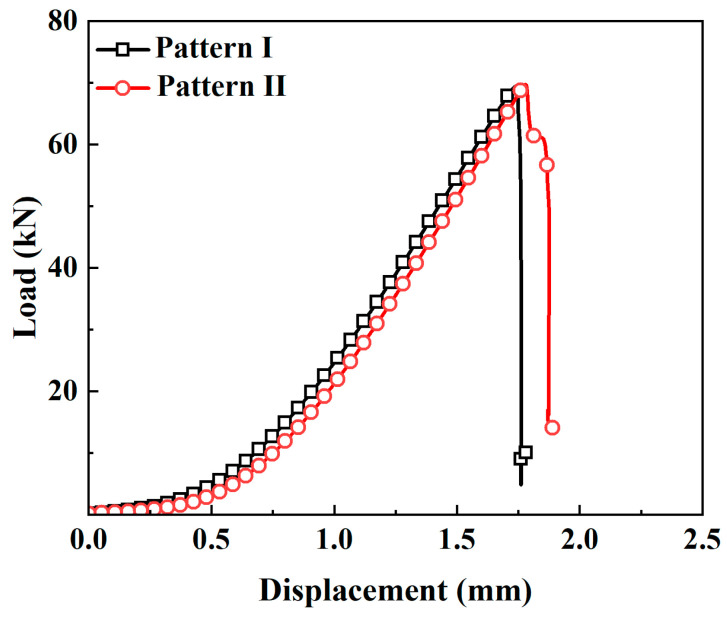
Two types of concrete splitting tensile load–displacement curves.

During the splitting tensile test of the rGFRP concrete specimens, the load–displacement curves exhibited two types of curve, as shown in Figure 11. The first type of curve corresponded to the load–displacement curves of all sample groups except C05. It was characterized by a rapid increase in load with an increase in displacement in the early loading stage. After reaching the peak load, the specimens cracked and were damaged rapidly, losing their load-bearing capacity instantaneously, followed by a rapid decrease in load until failure.

Furthermore, it was observed that the load–displacement curves exhibited by the concrete specimens of group C05 displayed distinctive behavior characterized by a second type of curve. The ascending portion of this type of curve shared similarities with that of the first type of curve. However, following the attainment of its peak, the load curve demonstrated a secondary peak after a period of decline. This phenomenon is often referred to as strain hardening. Subsequently, the load rapidly decreased until the concrete completely failed due to damage.

The underlying mechanism is as follows: When the applied load peaks and the concrete develops cracks, the stress is transferred to the fibers. Consequently, more fibers participate in the load-bearing process following cracking. The bond between these fibers and the matrix material creates an anchoring effect, thereby increasing the concrete’s load-bearing capacity. Simultaneously, some of the fibers break under the influence of the load, absorbing a certain amount of energy. However, when the fibers are pulled out or broken, they lose their load-bearing capacity, and the curve experiences a rapid descent.

Due to the limited length and number of fibers, the descending portion of the curve after reaching the peak is not gradual. This indicates that the reinforcing effect of these short fibers on the matrix is not optimal.

#### 3.2.4. Effect of rGFRP on the Microstructure of Concrete

**Figure 12:**
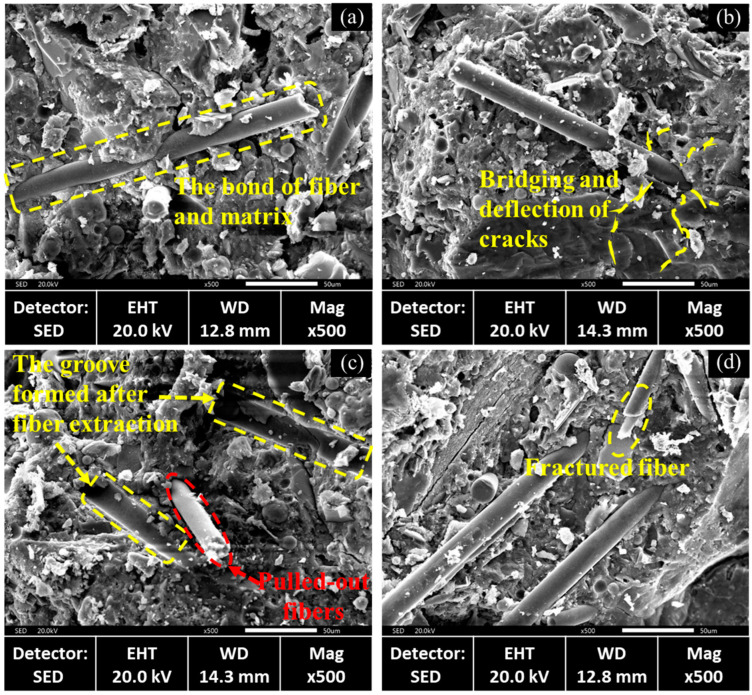
Enhancement mechanism of rGFRP in the base material.

To examine the mechanism of short fibers in rGFRP on the concrete microstructure, the small fragments that broke off during the compression and splitting tensile tests on rGFRP concrete were analyzed. The observation results are shown in Figure 12.

Upon examining the SEM images, it was observed that more fibers were pulled out than broken, suggesting that the bond between the fibers and the matrix was not robust, as shown in Figure 12a. As the fibers began to bear loads, a significant proportion of them slid without reaching their failure load, thereby failing to improve the strength of the concrete. The presence of fibers had a bridging and deflection effect on cracks, as shown in Figure 12b. As micro cracks propagated towards the fibers, these bridging and deflection effects prevented local stress concentration, making the concrete failure mode more flexible. As shown in Figure 12c, the fibers were pulled out, leaving visible grooves, and the fractured fiber is observed in Figure 12d.

#### 3.2.5. Effect of rGFRP on the Density of Concrete

**Figure 13:**
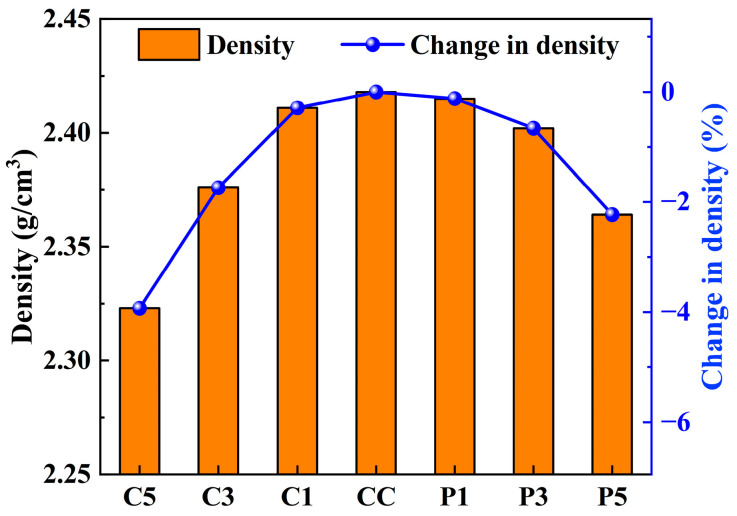
Effect of rGFRP on the density of concrete.

To investigate the effect of rGFRP on the density of concrete, the dry density of the concrete was calculated after 28 days of curing. To minimize errors, 12 concrete specimens were selected for density measurement in each group. The results, shown in Figure 13, indicate that the density of the control concrete was 2.42 g/cm^3^. As the amount of rGFRP increased, the density of the concrete decreased linearly. Since the density of rGFRP is lower than that of fine aggregate, increasing the content of rGFRP in concrete will lead to a reduction in the density of concrete.

When the rGFRP content was 3%, the compressive strength and splitting tensile strength of concrete improved, and the density of concrete reduced by 0.66% and 1.74%, respectively. Replacing 3% of fine aggregate with rGFRP in concrete appears to be a favorable option since it slightly increases strength while reducing density. When the rGFRP cluster content was 5%, the strength of the concrete decreased slightly, but the density of the concrete reduced by 3.93%, significantly decreasing the weight of the concrete structure. Therefore, in practical applications, determining the optimal dosage of rGFRP should be based on its intended use.

## 4. Optimization of Reusing rGFRP in Concrete

While some progress has been made in researching the mechanical recycling of rGFRP, its reuse in concrete has been primarily limited to laboratory research. Different scholars have focused on various aspects of this research, thus resulting in conclusions that may not be universally applicable. As a result, there appears to be a gap between the current state of research and its practical implementation in the industry. Yazdanbakhsh and Bank [42] believed that the main challenge in reusing rGFRP in concrete lies in the change in performance after its addition, rather than the uncertainty of recycled raw materials’ performance. The properties of rGFRP may significantly vary depending on its application and type. To achieve the industrialization of rGFRP concrete, it is essential to develop a comprehensive process to ensure its feasibility.

**Figure 14:**
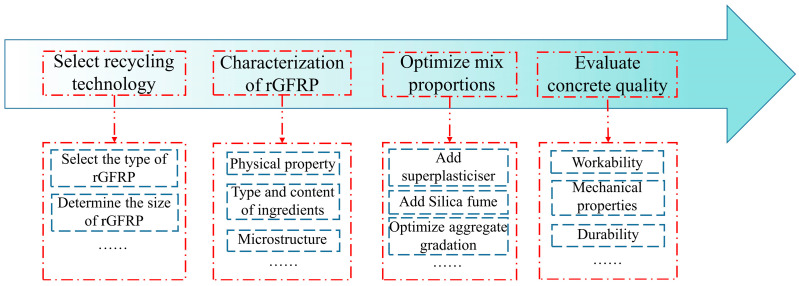
Optimization procedure for rGFRP reuse.

There is a lack of a widely applicable program for reusing GFRP. Building upon this study and other pertinent research, we propose an optimized rGFRP reuse program to address this gap. As shown in Figure 14, this program aims to establish a correlation between the properties of rGFRP concrete and the primary properties of rGFRP, defining the feasible range of rGFRP, and determine the optimal mix proportions for each rGFRP concrete through experimentation.

### 4.1. Selection of Recycling Technology

Currently, there are three ways to reuse rGFRP obtained through mechanical recycling in concrete. One approach involves processing GFRP waste into granules, which can serve as a fine aggregate replacement in concrete. Another method involves cutting GFRP waste into large pieces, suitable for use as a coarse aggregate replacement in concrete. Furthermore, GFRP waste can also be employed as fiber-shaped reinforcing bars by modifying the mechanical recycling processes. All three options for using rGFRP in concrete are attractive. Replacing aggregate comprising the largest proportion of concrete with rGFRP can maximize the reuse of GFRP recycled materials. Fiber-shaped rGFRP, retaining its relatively intact structure and performance, can be regarded as weakened fibers and has a significant enhancing effect on the mechanical properties of concrete.

Yazdanbakhsh et al. [43] conducted an experiment using FRP-RA made from waste GFRP rebar in concrete, partially replacing coarse aggregate. The results showed that the addition of FRP-RA reduces the strength of concrete.

Fu et al. [38] developed a new method for the mechanical recycling of GFRP waste by machining it into macro-fiber to reinforce concrete. The research demonstrated that adding macro-fibers improved the flexural strength and toughness of the concrete.

The results of prior studies in this research indicate that the impact of rGFRP on concrete properties varied according to the particle size levels. Likewise, Mastali et al. [44] observed that recycled GFRP fibers of varying lengths had diverse effects on the workability and mechanical properties of concrete. Various mechanical recycling processes can modify the shape and properties of rGFRP, affecting different aspects of concrete. Additionally, it is crucial to recognize that the cost of recycling GFRP waste is associated with the mechanical recycling process, highlighting the need to consider multiple factors when selecting a recycling method. Therefore, characterizing rGFRP accurately is crucial to identifying the most appropriate recycling process.

### 4.2. Characterization of rGFRP

Recycling plants that process waste from various sources of GFRP produce waste materials with different compositions. When incorporated into concrete, these waste materials can significantly affect the properties of the concrete, as shown in a study by García et al. [30], where significant differences in mechanical properties were observed in microconcrete prepared using rGFRP from four different sources. The unknown properties of rGFRP greatly constrain its potential for reuse. Therefore, it is essential to develop criteria and procedures for characterizing recycled GFRP waste from various sources.

The recycled waste should be tested to (a) measure its physical properties, (b) determine the type and content of its components, and (c) characterize its microstructure. Testing methods may include sieve analysis or laser particle size determination to determine particle size distribution, thermal analysis, and spectroscopic techniques such as XRD, XRF, and FT-IR to determine the type and content of fibers, resins, and other components in rGFRP, and scanning electron microscopy to determine the distribution of fibers and other components. Based on the data obtained from the tests and the properties of concrete containing GFRP waste, the usable range of the material can be determined.

Currently, there are limited studies in this area, and the performance of the raw materials used varies greatly. There is a lack of qualitative and quantitative understanding of the effects of the chemical composition and microstructure parameters of rGFRP on concrete performance.

### 4.3. Optimization of Mix Proportions

Mix proportion optimization aims to improve the performance of concrete and maximize the consumption of rGFRP while specifying performance requirements. Yazdanbakhsh and Bank [42] acknowledged that various forms of GFRP waste might negatively impact the performance of concrete. However, they believed that adjusting concrete mix proportions could resolve this issue. Previous research has shown that increasing the rGFRP content can significantly decrease concrete workability. However, increasing the w/c ratio to improve workability can result in decreased concrete strength. Therefore, multiple orthogonal mix proportion designs are required for optimizing mix proportions.

Varying mix proportions should be used for rGFRP concrete with different rGFRP content to ensure the desired workability and mechanical properties of the concrete, as well as to determine the optimal dosage of different types of rGFRP and their corresponding mix ratio. Optimization methods may include the use of superplasticizer, the addition of fly ash, adjustment of the w/c ratio, and the optimization of aggregate grading, among others.

With the development of computer technology, machine learning has been widely applied in the field of concrete mix design [45,46,47,48]. Compared to traditional design methods, machine learning can more accurately simulate the relationship between material variables and concrete performance to formalize the problem [49,50]. Therefore, using machine learning to design and optimize the mix proportion of rGFRP concrete is undoubtedly attractive, as it can greatly save time and reduce costs, all while achieving more accurate predictions of concrete performance [51].

### 4.4. Evaluation of Concrete Quality

The performance of rGFRP concrete exhibits significant variation due to the inherent variability in concrete's internal structure and the uneven distribution of rGFRP within it. So, it is essential to establish the quantitative relationship between factors such as the size and dosage of GFRP waste and the mechanical properties of concrete, which will determine their application in practical engineering projects. Currently, there is limited research in this direction, yet it is crucial because it can provide guidance for the mechanical recycling process of GFRP and the overall reuse of GFRP waste in concrete.

Apart from evaluating the workability and mechanical properties of concrete, it is crucial to emphasize its durability, especially in alkali–aggregate reaction testing. The alkali in cement can react with the silica in GFRP waste, leading to an alkali–silica reaction (ASR) that triggers destructive expansion and shortens the lifespan of concrete. Hence, thorough testing and evaluation of the alkali–aggregate reaction is necessary to ensure the durability and long-term performance of the rGFRP concrete. Moreover, feasible methods and technologies, such as adding mineral admixtures to suppress the alkali–aggregate reaction, need to be explored and applied to maximize the reliability and durability of rGFRP concrete.

## 5. Conclusions

GFRP products are increasingly being used in various industrial fields. However, the speed of GFRP waste accumulation is continuously increasing, which necessitates the development of more sustainable solutions within the GFRP industry. In light of this, this study conducted experiments to assess the reuse potential of recycled GFRP in concrete. The following conclusions were drawn from the experiments:

The addition of rGFRP reduced the slump of concrete. However, at lower content levels, the effect was negligible. 

The influence of a low content (1–5%) of rGFRP powder on the strength of concrete was minimal. The addition of a small amount of rGFRP cluster slightly improved the compressive strength and splitting tensile strength of concrete. The presence of short fibers in rGFRP improved the compressive and splitting tensile failure mode of concrete. Moreover, higher fiber content and longer fiber length exhibited a stronger reinforcing effect.

The addition of rGFRP decreased the density of concrete, indicating that it can somewhat facilitate the lightweight design of concrete structures.

To guarantee the quality and performance of concrete when using various GFRP waste materials, this study discussed and optimized existing reuse methods. It proposes an optimized procedure for GFRP reuse to maximize the technical and economic benefits. This procedure aims to facilitate the reuse of GFRP waste in engineering applications and advance its industrial-scale implementation.

Currently, there is a lack of understanding regarding the microstructural mechanisms underlying the impact of rGFRP on the mechanical properties of concrete. Further research is required to delve into this aspect, which will help advance the practical application of GFRP waste in engineering projects.

## Figures and Tables

**Table 1:** The chemical composition of raw materials according to XRF analysis (wt%).

Components	Na_2_O	MgO	Al_2_O_3_	SiO_2_	P_2_O_5_	SO_3_	K_2_O	CaO	Fe_2_O_3_	LOI
Cement	0.2	2.1	5.3	19.9	0.1	2.4	0.8	61.4	2.8	4.4
Fly ash	0.9	1.0	31.1	54.0	0.7	0.7	2.0	4.0	4.2	-
Mineral powder	-	6.0	17.7	34.5	-	1.6	-	34.0	1.0	0.8
rGFRP	0.4	1.9	10.0	40.4	0.1	0.1	0.4	19.9	0.8	25.4

**Table 2:** Mix proportion of rGFRP concrete (kg/m^3^).

Concrete Mixture	rGFRP Type	CA	FA	Cement	Superplasticizer	Fly Ash	Mineral Powder	Water	rGFRP
CC	--	1011	732	333	7.1	71.4	71.4	182	0
P01	powder	1011	725	333	7.1	71.4	71.4	182	7
P03	powder	1011	710	333	7.1	71.4	71.4	182	22
P05	powder	1011	695	333	7.1	71.4	71.4	182	37
C01	cluster	1011	725	333	7.1	71.4	71.4	182	7
C03	cluster	1011	710	333	7.1	71.4	71.4	182	22
C05	cluster	1011	695	333	7.1	71.4	71.4	182	37

**Table 3:** Summary of tests performed, specifications, and specimen sizes.

Test		Specification	Specimen Size
Fresh state	Slump	GB/T 50080 [35]	--
Hardened state	Compressive strength	GB/T 50081 [36]	100 × 100 × 100 mm^3^
	Tensile splitting strength	GB/T 50081 [36]	100 × 100 × 100 mm^3^
	Density	BS EN 12390-7 [37]	100 × 100 × 100 mm^3^

**Table 4:** Slump of fresh concrete (mm).

Concrete Mixture	Test No.							
	1	2	3	4	5	6	Mean	SD
CC	220	229	210	214	215	207	216	7.83
P01	200	210	196	182	183	193	194	10.60
P03	170	159	150	173	180	155	165	11.60
P05	68	51	80	54	39	72	61	15.25
C01	180	166	156	176	193	183	176	13.06
C03	114	107	143	142	126	122	126	14.60
C05	28	43	36	69	35	67	46	17.45

**Table 5:** Compressive strength of rGFRP concrete (MPa).

Concrete Mixture	rGFRP Content (%)	7-Day Compressive Strength (MPa)					28-Day Compressive Strength (MPa)				
		Specimen No.			Mean	SD	Specimen No.			Mean	SD
		1	2	3			1	2	3		
CC	0	33.45	34.15	37.17	34.92	1.97	45.17	40.43	43.92	43.17	2.45
P01	1	38.15	37.37	37.15	37.55	0.52	43.63	41.58	42.95	42.72	1.04
P03	3	32.67	32.23	30.55	31.82	1.12	45.40	44.61	40.33	43.45	2.73
P05	5	31.93	30.65	30.43	31.01	0.81	46.31	44.61	40.27	43.73	3.12
C01	1	30.30	30.16	34.39	31.62	2.40	49.61	47.55	41.16	46.11	4.41
C03	3	32.23	36.22	33.70	34.05	2.02	43.02	44.24	37.76	41.67	3.45
C05	5	34.89	32.93	32.11	33.31	1.43	39.38	37.58	38.61	38.52	0.91

**Table 6:** Splitting tensile strength of rGFRP concrete (MPa).

Concrete Mixture	rGFRP Content (%)	7-Day Splitting Tensile Strength (MPa)					28-Day Splitting Tensile Strength (MPa)				
		Specimen No.			Mean	SD	Specimen No.			Mean	SD
		1	2	3			1	2	3		
CC	0	2.86	2.92	3.30	3.03	0.24	3.63	4.23	3.60	3.82	0.36
P01	1	3.05	3.12	2.99	3.05	0.07	3.35	4.21	4.02	3.86	0.45
P03	3	2.90	2.86	2.92	2.89	0.03	4.30	3.86	3.78	3.98	0.28
P05	5	2.73	2.62	2.93	2.76	0.16	3.83	4.09	3.93	3.95	0.13
C01	1	3.20	2.99	2.98	3.06	0.12	3.34	4.10	3.92	3.79	0.40
C03	3	3.37	3.04	3.62	3.34	0.29	3.77	4.29	3.76	3.94	0.30
C05	5	2.73	3.11	2.89	2.91	0.19	3.65	3.26	3.54	3.48	0.20

## Data Availability

The data are contained within this article.
